# Diagnostic Utility of Ocular Symptoms and Vision for Cytomegalovirus Retinitis

**DOI:** 10.1371/journal.pone.0165564

**Published:** 2016-10-27

**Authors:** Yingna Liu, Alexander S. Chen, Siripim Kamphaengkham, Prattana Leenasirimakul, Choeng Jirawison, Somsanguan Ausayakhun, Todd P. Margolis, Jeremy D. Keenan

**Affiliations:** 1 Harvard Medical School, Boston, Massachusetts, United States of America; 2 Francis I. Proctor Foundation, University of California San Francisco, San Francisco, California, United States of America; 3 Yale University, New Haven, Connecticut, United States of America; 4 Department of Internal Medicine, Nakornping Hospital, Chiang Mai, Thailand; 5 Department of Ophthalmology, Nakornping Hospital, Chiang Mai, Thailand; 6 Department of Ophthalmology, Faculty of Medicine, Chiang Mai University, Chiang Mai, Thailand; 7 Department of Ophthalmology and Visual Sciences, Washington University School of Medicine, St. Louis, Missouri, United States of America; 8 Department of Ophthalmology, University of California San Francisco, San Francisco, California, United States of America; University of Florida, UNITED STATES

## Abstract

**Purpose:**

Cytomegalovirus (CMV) retinitis remains a leading cause of blindness in countries with a high burden of AIDS. Although dilated fundus examinations are recommended for those with CD4 counts below 100 cells/μL, in practice only those with poor vision and/or symptoms are routinely referred for screening. Therefore, the predictive value of this common practice should be assessed.

**Methods:**

This is a prospective cross-sectional study. Patients with known HIV and a CD4 count of less than 100 cells/μL attending an HIV clinic in Chiang Mai, Thailand completed a standardized questionnaire about visual symptoms and underwent visual acuity testing and dilated fundus examination. Participants without CMV retinitis were invited for repeated examinations every 3 months until their CD4 count exceeded 100 cells/μL. Patient-level statistical analyses were conducted to calculate diagnostic test characteristics, with bootstrapping to account for correlated data.

**Results:**

Of 103 study participants, 16 had CMV retinitis diagnosed at some point during the study. Participants with CMV retinitis were more likely to complain of visual symptoms compared to those without CMV retinitis (p = 0.01), including scotoma (p = 0.0002), itchy or watery eyes (p < 0.0001), and eye pain (p = 0.003); they were also more likely to have visual acuity worse than Counting Fingers (p = 0.0003). However, the absence of eye symptoms and the absence of poor vision did not strongly affect the probability that a patient did not have disease (negative likelihood ratio 0.56 and 0.76, respectively).

**Conclusions:**

Ocular symptoms and poor visual acuity were poor diagnostic indicators for the presence of CMV retinitis. Systematic screening of HIV patients with CD4 counts below 100 cells/μl should be carried out to detect disease at an early stage, when blindness can still be prevented.

## Introduction

Cytomegalovirus (CMV) retinitis is an opportunistic infection that is a leading cause of blindness in developing countries with a high burden of AIDS [1,2]. Experts generally recommend asymptomatic screening with indirect ophthalmoscopy for HIV patients with CD4 counts less than 100 cells/μl in order to diagnose the disease at an early stage before any visual disability has occurred. However, in resource-limited settings, the reality is that only patients with visual symptoms or poor visual acuity are referred for a screening examination. We were interested in assessing this common practice specifically for at-risk patients in a primary care setting in Asia. The predictive value of symptoms and vision in such a population has not been well characterized, even though the vast majority of CMV retinitis occurs in Asia [3], and even though primary care HIV providers make clinical decisions about whether to screen for CMV retinitis [1]. In this prospective cross-sectional study, we assessed the relationship between self-reported ocular symptoms, visual acuity, and an eventual diagnosis of CMV retinitis to determine whether HIV providers could increase the yield of eye screening examinations by asking about visual symptoms and testing for vision.

## Materials and Methods

This was a prospective cross-sectional study conducted with approval from the Committee on Human Research at the University of California, San Francisco and the Institutional Review Board of Nakornping Hospital, Chiang Mai, Thailand. It was performed in adherence with the tenets of the Declaration of Helsinki.

Details of the study population and enrollment process have been described elsewhere [4]. Briefly, from June 18, 2010 through June 15, 2012, patients with a CD4 cell count of less than 100 cells/μL who presented to the HIV clinic at Nakornping Hospital in Chiang Mai, Thailand were offered enrollment in the study. Patients who were pregnant, younger than 18 years, or already had a known diagnosis of CMV retinitis were excluded. After written informed consent, participants were asked whether they had any ocular symptoms using a standardized questionnaire administered by a designated nurse. Snellen visual acuity was then assessed with spectacles and pinhole, followed by a dilated fundus examination by a fellowship-trained retina specialist (CJ) to determine the presence or absence of CMV retinitis. Study participants without CMV retinitis were offered repeated screening every 3 months until their CD4 cell count increased to 100/μL or greater; the same questionnaire and examinations were conducted at each study visit.

We performed patient-level statistical analyses. Diagnostic test characteristics (i.e. sensitivity, specificity, positive and negative predictive values, positive and negative likelihood ratios), were calculated considering a positive test to be the presence of symptoms in either eye or the inability to count fingers (CF) at 1 meter in either eye, and patients were considered to have CMV retinitis if it was detected in either eye by the retina specialist. Study participants were censored after the first eye was diagnosed with CMV retinitis. Bootstrapped 95% confidence intervals (10,000 repetitions, re-sampled at the patient level) were calculated to account for correlated data (i.e., multiple visits by the same participant). All statistical analyses were performed using Stata 14.0 (StataCorp LP, College Station, Texas).

## Results

Of 258 patients with CD4 counts under 100 cells/μL seen in the HIV clinic during the study period, 103 were enrolled, including 23 who had 1 subsequent ophthalmologic examination and 5 who had 2 subsequent examinations. Mean age was 37.5 years and 61.2% were male. Mean enrollment CD4 count was 29.5 cells/μL. Sixteen patients were diagnosed with CMV retinitis in either eye at some point during the study.

Of the 16 person-visits in which CMV retinitis was diagnosed in either eye, ocular symptoms were reported beforehand in 9 (56.3%) and a visual acuity of worse than CF in the worse-seeing eye was detected in 4 (25.0%, Table 1). Of the 120 person-visits in which CMV retinitis was not detected in either eye, the corresponding numbers were 26 (21.7%) and 2 (1.7%, Table 1). Symptoms were more often reported at patient-visits in which CMV retinitis was diagnosed compared with those visits where CMV retinitis was not diagnosed (p = 0.01); symptoms that were significantly more common among patients with CMV retinitis included scotoma (p = 0.0002), itchy or watery eyes (p < 0.0001), and eye pain (p = 0.003). Reduced visual acuity was also more frequent when CMV retinitis was eventually diagnosed; patients diagnosed with CMV retinitis were more likely to have visual acuity worse than CF (p = 0.0003) and less likely to have visual acuity better than 20/40 compared to other patients (p = 0.02, Table 1).

**Table 1:** Association of cytomegalovirus (CMV) retinitis with ocular symptoms and visual acuity.

Characteristic	Number (%) or Mean (95% Confidence Interval)		*P*-value^b^
	No CMV retinitis N = 120 person-visits^a^	CMV retinitis N = 16 person-visits	
Symptoms			
Any symptom	26 (21.7%)	9 (56.3%)	0.01
Blurry vision	21 (17.5%)	3 (18.8%)	0.93
Flashes/floaters	9 (7.5%)	2 (12.5%)	0.48
Scotoma	1 (0.83%)	2 (12.5%)	0.0002
Itchy or watery eyes	1 (0.83%)	2 (12.5%)	<0.0001
Eye pain	1 (0.83%)	1 (6.25%)	0.003
Visual acuity			
Better than 20/40	106 (88.3%)	10 (62.5%)	0.02
20/40 to Counting Fingers	10 (8.3%)	2 (12.5%)	0.56
Worse than Counting Fingers	2 (1.7%)	4 (25.0%)	0.0003

^a^ Includes (1) the baseline examinations of 87 participants who were never diagnosed with CMV retinitis in either eye and 3 participants eventually diagnosed with CMV retinitis at the 3-month examination, (2) the 3-month examinations of 25 participants who were never diagnosed with CMV retinitis, and (3) the 6-month examinations of 5 participants who were never diagnosed with CMV retinitis in either eye.

^b^ Logistic regression performed with bootstrapped 95% confidence intervals to account for multiple visits from the same patient (10,000 replications, re-sampled at patient level)

Relative to an ophthalmologist examination, the presence of any ocular symptom had a sensitivity of 56.3% (95% confidence interval [CI] 32.0–80.5%) and specificity of 78.0% (95% CI 69.7–86.3%) for the diagnosis of CMV retinitis. In comparison, the presence of visual acuity worse than CF was 25.0% (95% CI 3.47–46.5%) sensitive and 98.3% (95% CI 95.9–100%) specific for diagnosing CMV retinitis (Table 2). In this study, the prevalence of CMV retinitis among the screened population was 15.5%. At this prevalence, the positive predictive value of ocular symptoms and visual acuity worse than CF were 25.7% (11.2–40.3%) and 66.7% (29.0–100%), respectively, and the corresponding negative predictive values were 92.9% (87.9–98.0%) and 90.6% (85.6–95.6%, Table 2). When diagnostic accuracy was expressed as likelihood ratios, the probability of having CMV retinitis was greatly increased by the presence of visual acuity worse than CF (positive likelihood ratio 14.8, 95% CI 2.93–74.2), whereas eye symptoms increased the probability of CMV retinitis by a smaller degree (positive likelihood ratio 2.6, 95% CI 1.5–4.4). The absence of symptoms and the absence of poor vision were much less useful for ruling out disease, with negative likelihood ratios of 0.56 (95% CI 0.32–0.99) and 0.76 (95% CI 0.57–1.01), respectively.

**Table 2:** Diagnostic test characteristics of ocular symptoms and visual acuity for the prediction of cytomegalovirus retinitis.

	Diagnostic test characteristic (95% Confidence Interval)^a^	
	Presence of any type of ocular symptom in either eye	Vision worse than Counting Fingers in the worse-seeing eye
Sensitivity	56.3% (32.0–80.5%)	25.0% (3.47–46.5%)
Specificity	78.0% (69.7–86.3%)	98.3% (95.9–100%)
Positive Predictive Value	25.7% (11.2–40.3%)	66.7% (29.0–100%)
Negative Predictive Value	92.9% (87.9–98.0%)	90.6% (85.6–95.6%)
Positive likelihood ratio	2.6 (1.5–4.4)	14.8 (2.93–74.2)
Negative likelihood ratio	0.56 (0.32–0.99)	0.76 (0.57–1.01)
ROC area	0.67 (0.54–0.80)	0.62 (0.51–0.73)

ROC: receiver operating characteristic

^a^ Bootstrapped 95% confidence intervals (CIs) constructed (10,000 replications, sampled at the patient level) to account for multiple visits from the same individual.

## Discussion

In this study of HIV-positive patients with a CD4 count below 100 cells/μL presenting to a primary care HIV clinic, the presence of ocular symptoms and reduced visual acuity considerably increased the likelihood of having CMV retinitis (i.e., high positive likelihood ratio), but had low positive predictive value at the relatively low prevalence of retinitis found in this population. The absence of eye symptoms and absence of poor vision were better predictors of not having CMV retinitis in this study population. However, the high negative predictive value of these tests was also likely due to the low prevalence of disease, since the absence of eye symptoms and poor vision did not greatly change the likelihood of whether an individual had CMV retinitis (i.e., low negative likelihood ratio).

Although several prior studies have assessed the relationship between visual symptoms, visual acuity, and CMV retinitis, few have been done in Asia [5]. The most relevant prior studies are from India and Vietnam, and enrolled prospective series of HIV patients for CMV retinitis screening [6,7]. The present study differs from these two studies in that unlike the Indian study, we included only those HIV patients with a CD4 count less than 100 cells/μL, and unlike the Vietnamese study, we performed screening at a primary care HIV clinic instead of an eye clinic. Our findings were generally consistent with these other two studies, which also found a poor positive predictive value (18–40%) and a higher negative predictive value (40–95%).

Some previous studies have assessed the relationship between milder forms of visual impairment and CMV retinitis [7,5]. However, we thought it was unlikely that providers in a busy HIV clinic would routinely test for Snellen visual acuity. We reasoned that testing for Counting Fingers vision was a quick test that could easily be incorporated into an HIV provider’s clinic visit, and hence could be a valuable diagnostic test. We found that this test for low vision was a poor predictor for CMV retinitis. Its negative predictive value was better; that is, those with vision of Counting Fingers or better were unlikely to have CMV retinitis. However, the high negative predictive value was most likely due to the overall low prevalence of CMV retinitis in the population, since poor vision had a negative likelihood ratio of 0.76, which suggests that having good vision did not strongly change the probability that a person did not have CMV retinitis.

Our study also found that CMV retinitis patients were more likely to complain of itchy or watery eyes compared to HIV patients without CMV retinitis. Keratoconjunctivitis sicca, which was previously one of the most common ophthalmic manifestations of HIV/AIDS, is thought to be caused by a Sjogren-like immune dysfunction and abnormal lymphocytic infiltration of the lacrimal gland [8]. The prevalence of dry eye syndrome among HIV patients in the United States has significantly decreased over the years—possibly as a result of more widespread access to antiretroviral therapy [9]. The results of our study suggest that keratoconjunctivitis sicca may still occur in a minority of immunocompromised HIV patients in Asia, and that dry eyes may indicate a higher risk of CMV retinitis. We can only speculate as to whether any immune dysfunction associated with dry eye disease also makes one more likely to develop CMV retinitis. Regardless, we conclude that while scotoma, floaters, and flashes are important symptoms because of their association with vision-threatening retinitis lesions and retinal detachment, dry eye symptoms are also important to elicit during history-taking.

We specifically designed the present study to mimic the clinical decision-making that an HIV provider faces when deciding whether to perform CMV retinitis screening. We reduced selection bias by conducting the study at a primary care HIV clinic instead of an ophthalmology clinic and by enrolling only those patients with CD4 counts below 100 cells/μL, who are at the greatest risk. We performed the study prospectively to reduce the chances of misclassification bias. The study’s chief limitations were the relatively low numbers of patients diagnosed with CMV retinitis and uncertain generalizability outside Thailand.

In conclusion, this prospective cross-sectional analysis showed that ocular symptoms and poor visual acuity were poor diagnostic indicators for the presence of CMV retinitis. Routine, systematic screening of HIV patients with CD4 counts below 100 cells/μl is important for detecting disease at an early stage, when appropriate treatment can still prevent blindness.

## Supporting Information

S1 File This is the data set used for this study. (XLSX)
